# Impact of a health marketing campaign on sugars intake by children aged 5–11 years and parental views on reducing children’s consumption

**DOI:** 10.1186/s12889-020-8422-5

**Published:** 2020-03-30

**Authors:** Jennifer Bradley, Grace Gardner, Maisie K. Rowland, Michaela Fay, Kay Mann, Richard Holmes, Emma Foster, Catherine Exley, Ann Don Bosco, Orla Hugueniot, Paula Moynihan

**Affiliations:** 1 Human Nutrition Research Centre, Population Health Sciences Institute, Faculty of Medical Sciences, Newcastle University, Framlington Place, Newcastle upon Tyne, NE2 4HH UK; 2 School of Dental Sciences, Newcastle University, Framlington Place, Newcastle upon Tyne, NE2 4BW UK; 3 Population Health Sciences Institute, Faculty of Medical Sciences, Newcastle University, Framlington Place, Newcastle upon Tyne, NE2 4HH UK; 4 The Australian e-Health Research Centre, CSIRO, Brisbane, QLD 4029 Australia; 5 Public Health England, 133 – 155 Waterloo Road, London, SE1 8UG UK; 6 Adelaide Dental School, Faculty of Health and Medical Sciences, The University of Adelaide, Adelaide, SA 5005 Australia

**Keywords:** Diet, Sugars, Children, Health marketing

## Abstract

**Background:**

The association between Free Sugars intake and non-communicable diseases such as obesity and dental caries is well documented and several countries are taking measures to reduce sugars intakes. Public Health England (PHE) instigated a range of approaches to reduce sugars, including a national health marketing campaign (Sugar Smart). The campaign aimed to raise awareness of the amount of sugars in foods and drinks and to encourage parents to reduce their children’s intake. The aim of this study was to determine whether the campaign was effective in altering dietary behaviour, by assessing any impact of the campaign on sugars intake among children aged 5–11 years. Parental perceptions of the campaign and barriers to reducing sugars intake were also explored.

**Methods:**

Parents of 873 children aged 5–11 years, identified from an existing PHE database, were invited to take part. Dietary information was collected online using Intake24 before, during, and at 1, 10 and 12 months following the campaign. Change in sugars intake was assessed using mixed effects linear regression models. One-to-one telephone interviews were conducted with a purposive sample of parents to explore perceptions of the campaign and identify barriers and facilitators to reducing children’s sugars intake.

**Results:**

Completion rates for dietary assessment ranged from 61 to 72% across the follow up time points. Qualitative telephone interviews were conducted with 20 parents. Total sugars intake decreased on average by ~ 6.2 g/day (SD 43.8) at peak campaign and the percentage of energy from total sugars significantly decreased immediately and 1 year post campaign. The percentage of energy from Free Sugars significantly decreased across all time points with the exception of the long term follow up at 12-months post campaign. The percentage of energy intake from total fat increased. Parents expressed a willingness to reduce sugars intakes, however, identified barriers including time constraints, the normalisation of sugary treats, and confusing information.

**Conclusions:**

A health marketing campaign had a positive impact in reducing sugars intake but reductions in sugars were not sustained. Parents want to reduce their child’s sugars intake but societal barriers and confusion over which sources of sugars to avoid hamper efforts to change.

## Background

The association between Free Sugars intake and non-communicable diseases (NCDs) such as obesity and dental caries has been well documented [1–4] and there is a wealth of evidence suggesting current sugars intakes are exceeding recommendations in many countries [5–7]. Free Sugars include all mono- and di-saccharides added to foods by manufacturer, cook or consumer, plus those sugars naturally present in honey, syrups, fruit juices and fruit juice concentrates^1^ [8].

In 2015 the World Health Organisation issued a strong recommendation that Free Sugars intakes by individuals should not exceed 10% of total energy intake, with a conditional recommendation to reduce Free Sugars intake to below 5% of total energy intakes [9]. Following this, the Scientific Advisory Committee on Nutrition (SACN) recommended that at a population level, the intake of Free Sugars should not exceed 5% of total energy intake in the UK [10]. Likewise, in the US Department of Agriculture recommend that Americans should consume less than 10% of calories as added sugars [11] (which includes syrups and honey, but excludes those Free Sugars in fruit juices and fruit juice concentrates).

Data from the UK National Diet and Nutrition Survey (NDNS) indicate that intake of Free Sugars by all age groups exceeds the recommendation [12]. Boys and girls aged 4–10 years received an average of 13.6 and 13.4% (respectively) of their food energy from Free Sugars. Only 3% of boys and 1% of girls had intakes below or equal to 5% total energy. In the US more than 13% of energy is provided by added sugars [11], and only 33% of children aged 2–19 years meet the dietary guideline recommendation [6].

Considerable action, including upstream and downstream preventive measures is therefore required to bring sugars intake in line with current recommendations. Following a review of the evidence for action on sugar reduction, including what drives excessive sugars intake, PHE proposed a broad range of measures to reduce sugars intake [13]. Actions included upstream approaches, such as the introduction of a structured, transparent programme of sugar reduction and a wider reformulation programme. With all sectors of the food industry challenged to reduce the overall sugars content of key foods that contribute to intakes of children by around 20% by 2020 through product reformulation to cut the sugars levels in products; a reduction in portion size; and/or a shift in consumer purchasing towards lower/no-added sugars products and introducing a levy on sugar-sweetened beverages [13]. However, if instigated without first building public support such measures can attract criticism for interfering unduly with personal choice [14–16].

Downstream approaches aim to inform the public, change opinion and build support for change. PHE proposed to reduce sugars intake by raising awareness of the amount of sugars in children’s diets in England in comparison with government recommendations, problems around high amounts of sugars in the diet and to encourage families to take action to reduce intakes [13]. In 2016, PHE launched the Change4Life Sugar Smart Campaign [17]. This health marketing campaign used TV, radio, a digital product and advertising to: raise awareness of the high levels of sugar consumed by children and the associated health harms; raise awareness of the amount of added sugars in everyday foods and drinks; and to encourage parents to cut down the amount of such sugars their children consumed.

Sugar Smart packs were distributed to primary age children via schools, which provided families with information about guidelines for sugars intake and practical information to help them reduce sugars in their children’s diet. A free Sugar Smart app was available to download, which enabled parents to see how much sugars was contained in everyday foods and drinks (depicted in sugar cubes) by scanning the barcode on pack.

The main purpose of health marketing campaigns such as the Change4Life Sugar Smart Campaign is to raise awareness, change attitudes and ultimately help shift the behaviour of the population. Information on the impact on attitudes and on dietary behaviour is therefore important in determining if health marketing is an effective means of helping to reduce a populations sugars consumption.

The aim of the present study was to assess any impact of the Change4Life Sugar Smart Campaign on the dietary behaviour of children aged 5–11 years whose parents had shown an interest in previous Change4Life campaigns. A second aim was to explore any impact in awareness around sugars and to identify any potential barriers and facilitators to reducing sugars intake.

The objectives were:

- to measure the total intake of dietary sugars (type, amount g/day, percent contribution to total energy intake, and dietary sources) in a population of children aged 5–11 years (girls and boys from a range of socioeconomic, ethnic and family backgrounds);
- to assess any change in sugars intake during and after the Change4Life campaign;
- to obtain qualitative data from a sub-sample of parents about their understanding of the campaign messages, knowledge of sugars and any perceived barriers and facilitators to reducing their child’s sugar intake.

## Methods

### Participant recruitment

In liaison with ‘Kantar Public UK’ and PHE, participants were recruited from families that had registered with the PHE Change4Life database. In order detect a 10% change in sugars intake by both boys and girls with 90% power at the 0.05 alpha, the target sample size was 289 boys and 256 girls (a total of 545 with equal numbers from two socio-economic groups (A, B, C1 and C2, D, E [18])), from a range of ethnic backgrounds (White, Asian/Asian British, Black/African/Caribbean/Black British, mixed/multiple ethnic, other ethnic group) and geographic areas in England. To account for attrition (a potential loss to follow up of 42%, informed by the National Diet and Nutrition Survey (NDNS) [19]) a target sample size of 775 (411 boys and 364 girls) was set.

Kantar Public UK sent a recruitment email to parents including an online screening questionnaire to determine those who were eligible to take part in the study (i.e. the parent/guardian had at least one child aged 5–11 years). Eligible participants received an electronic participant information, consent (and child assent) documents which parents/ guardians were asked to complete online. Ethical approval was obtained from Newcastle University Ethics Committee (application number 01030).

### The sugar smart campaign

**Fig. 1:**
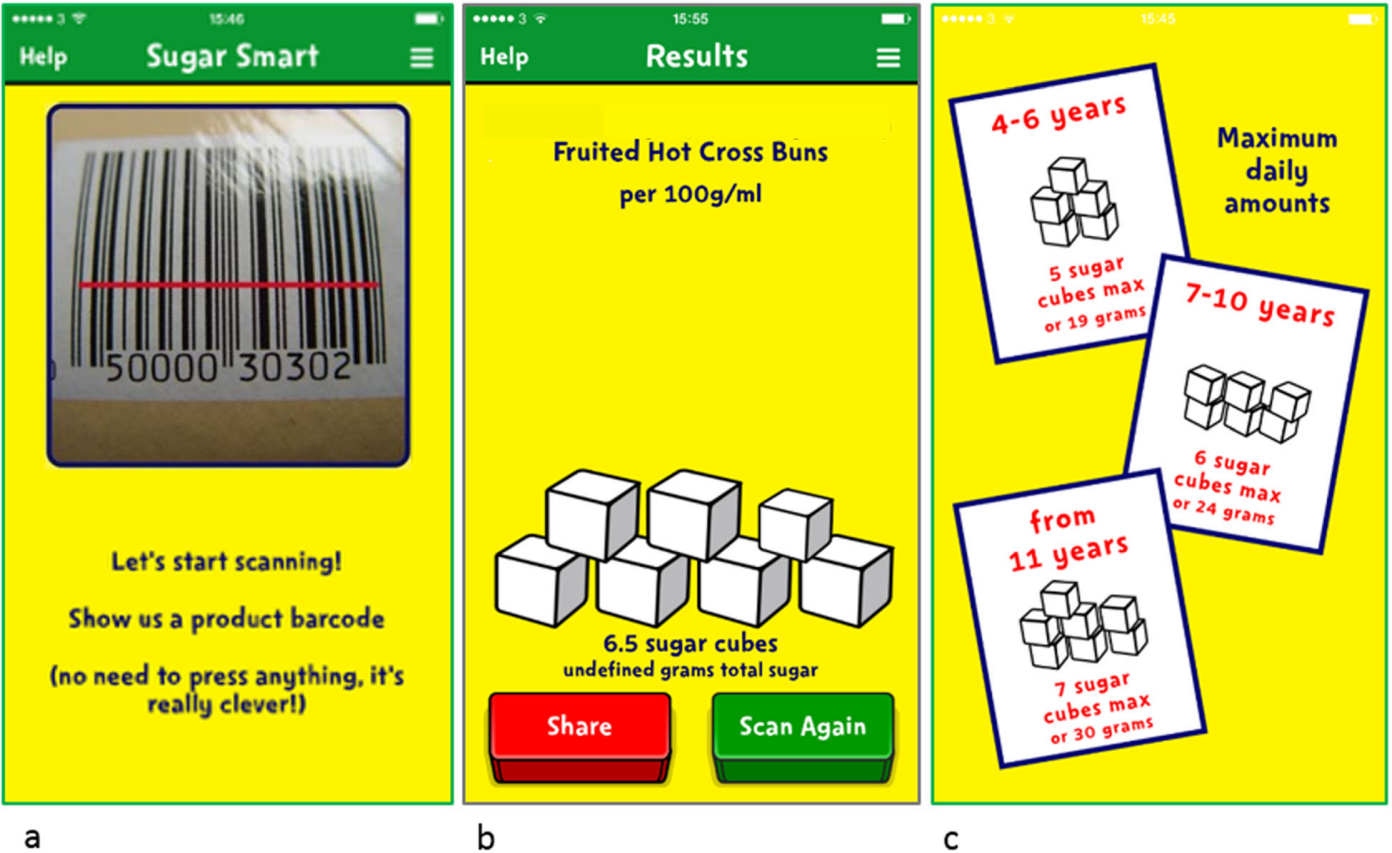
Screen shots from the Change4Life Sugar Smart app. **a** Barcode scanner on the Sugar Smart app. **b** Amount of total sugars contained in the food depicted in sugar cubes. **c** Information available on the app regarding maximum daily amounts of sugar for children

The Change4Life Sugar Smart campaign was launched on January 4th 2016. TV, billboard and digital advertising ran for 6 weeks to support the campaign. A Sugar Smart app was available for parents to download free of charge (Fig. 1). The app enabled users to scan barcodes on food and drink packaging to find out how many grams of total sugars were contained in the product; depicted in sugar cubes, to help consumers visualise the amount. Sugar Smart packs were distributed to primary school children, which provided children and parents with further information and tools to help them cut down on sugars. The pack informed on thresholds for sugars intake and a guide indicating the sugars content of popular foods and drinks, practical guidance on how to reduce sugars intake, and information about the Sugar Smart app. Further details about the campaign can be found at the Change4Life website [20].

### Dietary data collection using Intake24

Information on the dietary intake of each participating child was collected over 2 weekend days at five time points; baseline (2nd and 3rd January 2016), peak campaign (30th and 31st January 2016), immediately post campaign (27th and 28th February 2016), to identify any short-term effects of the campaign, and 10-months (20th and 21st November 2016) and 12-months post campaign (29th and 30th December 2016), to identify any sustained effects. To control for seasonal fluctuations in intakes (due to the timing of the campaign around the Christmas/New Year period), the long term follow up was exactly 12 months from baseline. Data were also collected at 10-months post campaign (outside the Christmas period).

Parents/guardians were asked to report their child’s dietary intake using an online self-completed 24-h dietary recall system ‘Intake24’ (https://intake24.co.uk) [21]. Parents/ guardians were assigned a unique username and password for Intake24 and were asked to log on and report everything their child had to eat and drink the day before. An email was sent to parents the day before the recall day to remind them to log on the next day to complete Intake24. Parents also received an email on each recall day to prompt them to complete. Intake24 uses a well-established multiple-pass recall method whereby the user records all food and drinks consumed in the previous 24 h [22]. Portion sizes were estimated using a database of over 2400 photographs of more than 100 foods. The Intake24 database converts foods and drink reported to average daily intake of nutrients (e.g. sugars) through integrated food composition tables. The dietary outcomes included amount of sugars as well as the contribution of sugars to energy (kJ) as the latter accounts for increase in amount of food intake with age over the year of study. The dietary variables therefore included: total sugars (g/day); NME Sugars (g/day) (as NMES is a proxy for Free Sugars) assessed using the NDNS method [23]; the percentage contribution of total sugars and of Free Sugars to total energy intake; the percentage contribution of main sources of sugars to total sugars intake; the change in intake of total energy and of total fat intake were also calculated.

Reported energy intake values were validated using parent-reported child body weight to determine basal metabolic rate [24] which was compared with reported energy intake to derive a Physical Activity Level (PAL). The Torun cut off values for PAL were used to identify any underreporting [25].

### Statistical analysis

Mean intakes of energy (kJ), total sugars (g and % energy), Free Sugars (g and % energy) and fat (g and % energy) at each of the follow up time points were compared with baseline. Mixed effects linear regression models were used to assess changes in intakes over time, with a random effect at the participant level to account for repeated measurement on the same individual. To consider individual variation in changes in intakes over time, a random intercept with random slope model was used. Models were subsequently adjusted for gender and socioeconomic group. All individuals were included in the regression model as missing data can be handled within this framework using maximum likelihood estimation on available data at each time point. All analyses were conducted using Stata 15 (StataCorp, College Station, Texas, USA).

### Qualitative interviews and analysis

One-to-one semi-structured qualitative interviews were conducted by telephone with a purposive sample of parents directly following the launch of the campaign. Parents who had indicated at recruitment that they were willing to take part in a telephone interview and who had completed dietary recalls for their child were contacted by email from the research team inviting them to participate in an interview. Sampling took into consideration child age, head of household occupation and geographical location.

Interviews explored a number of topics including parents’: 1) understandings of the messages of the Change4Life campaign; 2) understandings of sugars and its impacts on health; and 3) accounts of the individual, family and social barriers experienced with respect to reducing their child’s sugars intake.

Each interview was digitally recorded, transcribed verbatim and thematically coded and analysed using NVivo qualitative data analysis Software, version 11 (QSR International Pty Ltd.).

## Results

In total, 837 participants were invited to take part in the first three time points. For the long-term follow-up, the 539 participants who completed two baseline dietary recalls, were invited to take part. The completion rates and sample demographics for each time point of the study are shown in Table 1.

**Table 1:** Completion rates and sample characteristics of those included in the analysis at each time point

	Baseline (*n* = 590)	Peak-campaign (*n* = 553)	Immediately post campaign (*n* = 497)	10-months post campaign (*n* = 371)	12-months post campaign (*n* = 380)
Number of participants contacted to take part	837	837	837	539	539
Number of participants completing at least one dietary recall	602	570	506	372	381
Completion rate (%)	71.9	68.1	60.5	69.0	70.7
Participants removed from dataset due to invalid dietary recalls (n)	12	17	9	1	1
Number of participants included in analysis	590	553	497	371	380
Age in years (n [%])					
5	144 (24)	132 (24)	119 (24)	84 (23)	82 (22)
6	91 (15)	89 (16)	75 (15)	55 (15)	57 (15)
7	85 (14)	79 (14)	70 (14)	57 (15)	58 (15)
8	86 (15)	78 (14)	66 (13)	53 (14)	53 (14)
9	70 (12)	68 (12)	58 (12)	44 (12)	47 (12)
10	68 (12)	65 (12)	63 (13)	44 (12)	48 (13)
11	46 (8)	42 (8)	46 (9)	34 (9)	35 (9)
Mean age (years) [Standard deviation]	7.4 [2.0]	7.4 [2.0]	7.5 [2.0]	7.5 [2.0]	7.6 [2.0]
Gender (n [%])					
Male	279 (47)	257 (46)	234 (47)	172 (46)	175 (46)
Female	311 (53)	296 (54)	263 (53)	199 (54)	205 (54)
Ethnicity (n (%))					
White	529 (90)	493 (89)	440 (89)	331 (89)	336 (88)
Asian/Asian British	31 (5)	32 (6)	29 (6)	21 (6)	20 (5)
Black/African/Caribbean/Black British	12 (2)	11 (2)	12 (2)	8 (2)	9 (2)
Mixed/multiple ethnic	9 (2)	9 (2)	7 (2)	6 (2)	8 (2)
Other Ethnic Group	4 (1)	4 (1)	4 (1)	1 (0)	3 (1)
Prefer not to answer	5 (1)	4 (1)	5 (1)	4 (1)	4 (1)
Socioeconomic group (n [%])					
ABC1	396 (67)	370 (67)	339 (68)	252 (68)	255 (67)
C2DE	194 (33)	183 (33)	158 (32)	119 (32)	125 (33)

A small number of invalid recalls were eliminated from the final dataset at each time point. Reasons included, the inclusion of alcohol (possibility that the parent completed the recall for themselves instead of their child), or a recall time of less than 2 min (suggesting the recall had not been completed accordingly).

### Dietary outcomes

The average daily intakes of energy, sugars and fat at baseline, peak-campaign, immediately post-campaign, 10-months post campaign and 12-months post campaign are presented in Table 2.

**Table 2:** Mean (SD) and median (IQR) sugars and nutrient intakes

Nutrient	Short term						Long term			
	Baseline [*n* = 590]		Peak campaign [*n* = 553]		Immediately post campaign [*n* = 497]		10-month post campaign [*n* = 371]		12-month post campaign [*n* = 380]	
	Mean (SD)	Median (IQR)	Mean (SD)	Median (IQR)	Mean (SD)	Median (IQR)	Mean (SD)	Median (IQR)	Mean (SD)	Median (IQR)
% Energy from Total sugars	27.2 (8.5)	26.9 (15.6, 38.2)	25.2 (8.2)	24.7 (13.8, 35.6)	25.1 (8.5)	23.8 (13.7, 23.8)	24.2 (8.1)	23.7 (14.3, 33.1)	25.4 (7.9)	24.7 (14.0, 35.4)
Total sugars (g/day)	100.9 (46.4)	92.5 (34.4, 150.6)	93.9 (44.1)	87.1 (35.5, 138.7)	95.2 (41.5)	88.2 (39.1, 137.3)	95.9 (44.6)	90.5 (35.7, 145.3)	102.0 (46.0)	94.6 (38.9, 150.3)
% Energy from Free Sugars	16.1 (7.8)	15.2 (4.6, 25.8)	14.8 (7.8)	14.0 (3.2, 24.8)	15.1 (7.7)	14.0 (4.2, 23.8)	14.9 (7.5)	14.1 (5.0, 23.2)	16.3 (8.0)	15.3 (4.4, 26.2)
Free Sugars (g/day)	61.4 (38.9)	53.1 (7.0, 99.2)	56.5 (36.4)	50.3 (4.2, 96.4)	58.4 (35.3)	50.7 (7.4, 94.0)	60.5 (38.0)	54.5 (7.3, 101.7)	66.7 (40.4)	59.1 (4.8, 113.4)
% Energy from Fat	30.1 (6.5)	29.8 (21.4, 38.2)	31.4 (6.0)	31.5 (23.2, 39.8)	31.4 (6.2)	31.8 (23.6, 40)	32.3 (5.9)	32.2 (23.9, 40.5)	32.6 (6.1)	32.7 (24.5, 40.9)
Total fat (g/day)	52.1 (22.2)	48.4 (21.8, 75.0)	54.6 (21.2)	51.3 (25.1, 77.5)	56.3 (20.8)	52.7 (26.5, 78.9)	59.6 (23.3)	55.1 (23.5, 86.7)	61.6 (25.2)	57.1 (25.8, 88.4)
Energy (kJ/day)	6253.6 (1924.0)	6060.8 (3611.5, 8510.1)	6316.4 (1895.8)	6117.1 (3797.8, 8436.4)	6484.2 (1756.7)	6325.1 (4066.6, 8583.6)	6704.5 (2100.4)	6497.7 (3750.7, 9244.7)	6826.0 (2119.0)	6614.4 (4100.4, 9128.4)

Free Sugars were assessed as non-milk extrinsic sugars using method used in the National Diet and Nutrition Survey [23]

The PAL ratios averaged 1.35 (±0.41) and 1.50 (±0.44) for 5 year old and 1.27 (±0.36) and 1.40 (±0.47) for 6–11 year old boys and girls respectively. There was a statistically significant decrease in the percentage energy from total sugars across all time points compared with baseline, ranging from 2.5% at 10-months post campaign (*p* < 0.001), to 1.4% at 12-months post campaign (*p* < 0.001, Table 3). A significant decrease in the amount (grams per day) of total sugars consumed was seen at peak campaign (by 6.2 g/day, *p* < 0.001), immediately post campaign (by 5.5 g/day, *p* = 0.002), and 10-months post campaign (3.5 g/day, *p* = 0.03). Percentage energy from Free Sugars (NMES) significantly decreased across all time points with the exception of 12-months post campaign. The percentage energy from fat increased significantly across all time points, with the largest increase at 12-months post-campaign (2.4%, *p* < 0.001). Energy intakes significantly increased across all the post-campaign time points. Adjustment for gender and socioeconomic group (ABC1 and C2DE) did not attenuate the change in intakes across any time point.

**Table 3:** Post campaign changes in sugars and nutrient intakes

	Baseline-peak campaign			Baseline-immediately post campaign			Baseline-10 months post campaign			Baseline-12 months post campaign		
Nutrient	Mean change in intake (SD)	95% CI	*p*-value	Mean change in intake (SD)	95% CI	*p*-value	Mean change in intake (SD)	95% CI	*p*-value	Mean change in intake (SD)	95% CI	*p*-value
% Energy from Total sugars	−1.9 (8.7)	−2.6, −1.2	< 0.001	−2.0 (9.2)	−2.7, −1.3	< 0.001	−2.5 (9.8)	−3.5, − 1.9	< 0.001	− 1.4 (9.1)	− 2.5, −0.9	< 0.001
Total sugars (g/day)	−6.2 (43.8)	−9.5, − 2.9	< 0.001	−5.5 (43.2)	−9.0, − 2.0	0.002	− 3.5 (50.0)	−8.8, − 0.6	0.03	2.0 (52.2)	− 3.2, 5.5	0.61
% Energy from Free Sugars	− 1.2 (8.1)	−1.8, − 0.6	< 0.001	− 1.0 (8.2)	−1.5, − 0.24	0.007	−0.9 (8.9)	− 1.8, − 0.32	0.005	0.5 (8.9)	−0.5, 1.0	0.49
Free Sugars (g/day)	−4.1 (36.1)	− 6.8, − 1.2	0.005	−2.7 (37.0)	−5.5, 0.56	0.11	0.4 (42.7)	−4.1, 3.0	0.77	6.7 (45.5)	1.95, 9.5	0.003
% Energy from Fat	1.1 (6.9)	0.6, 1.7	< 0.001	1.2 (7.4)	0.6, 1.8	< 0.001	2.0 (7.6)	1.5, 2.7	< 0.001	2.4 (7.0)	1.8, 3.1	< 0.001
Total fat (g/day)	2.3 (22.4)	0.6, 4.5	0.01	4.0 (24.2)	1.9, 6.0	< 0.001	6.8 (26.8)	4.7, 9.5	< 0.001	9.5 (27.1)	6.9, 11.8	< 0.001
Energy (kJ/day)	− 69.3 (1801.9)	−71.9, 235.0	0.30	223.0 (1884.2)	51.5, 381.4	0.01	428.8 (2168.8)	225.2, 612.0	< 0.001	574.2 (2341.1)	350.8, 760.3	< 0.001

Short-term changes include baseline to peak-campaign and baseline to post-campaign, and long-term changes include baseline to 10-months post campaign and baseline to 12-months post campaign

Results from unadjusted linear regression models

### Sources of sugars

The percent contributions of the main dietary sources of sugars to total sugars intake are shown in Table 4. Data are presented for baseline, peak-campaign and 12-months post campaign (as indicators of short term and long term effect). Interquartile ranges indicate wide variation in the contribution of food groups to total sugars intake. There was a trend towards a small decrease in the contribution of fresh fruit to total sugars over the 12-month period and an increased contribution of soft drinks was observed. The contribution of other sources remained similar across the three time points.

**Table 4:** Percentage contribution (median (IQR)) of food groups to total sugars intake

Food Groups	% Contribution to total sugars intake		
	Baseline (*n* = 527)	Peak-campaign (*n* = 503)	12-months post campaign (*n* = 342)
	Median (IQR)	Median (IQR)	Median (IQR)
Fresh fruit	13.0 (0.0–26.5)	12.8 (3.3–24.8)	11.2 (0.0–21.4)
Soft drinks (not diet)	1.8 (0.0–18.9)	2.2 (0.0–16.4)	9.3 (0.0–29.6)
Fruit juice	0.0 (0.0–18.9)	0.0 (0.0–17.2)	0.0 (0.0–18.4)
Confectionery – sweets and chocolate	3.1 (0.00–12.7)	0.0 (0.0–10.3)	5.0 (0.0–16.0)
Cakes and biscuits	2.9 (0.0–9.7)	4.4 (0.0–11.8)	3.5 (0.0–10.7)
Breakfast cereals	3.2 (0.0–6.6)	3.4 (0.0–7.6)	2.5 (0.0–6.3)
Sugar, honey and preserves	0.0 (0.0–5.5)	0.0 (0.0–5.1)	0.0 (0.0–6.1)
Whole milk yoghurts/ fromage frais	0.0 (0.0–4.8)	0.0 (0.0–5.9)	0.0 (0.0–4.1)

*IQR* Interquartile Range

Data presented are for participants completing two recalls at each time point and include consumers and non-consumers

### Findings from qualitative interviews

The demographics of the sub-sample of participants who took part in the telephone interviews are shown in Table 5.

**Table 5:** Sample characteristics of those completing telephone interviews (*n* = 20)

	n (%)
Total number of children in household	
1	8 (40%)
2	5 (25%)
3	5 (25%)
4	2 (10%)
Gender of participating child	
Male	12 (60%)
Female	8 (40%)
Gender of interviewee	
Male	3 (15%)
Female	17 (85%)
Ethnicity	
White	14 (70%)
Asian/Asian British	3 (15%)
Black/African/Caribbean/Black British	2 (10%)
Other Ethnic Group	1 (5%)
Socioeconomic group	
ABC1	11 (55)
C2DE	9 (45)

The qualitative findings are summarised in Table 6. There was evidence of awareness raising of sugars consumption among parents and children, however, this was accompanied by an increased confusion over ‘good’ and ‘bad’ sugars. Parents described a number of behavioural changes following the campaign, including swapping ‘unhealthy’ foods for healthier versions or for a different product, and reducing portion sizes. Parents highlighted several barriers which prevent dietary change. These included time constraints due to busy lifestyles, leniency with regard to permitting sugary treats, and peer pressure from other parents who allow their children to consume sugary foods and drinks. Parents also described the easy availability of sweet treats and snacks not only in supermarkets but also in schools in the form of puddings.

**Table 6:** Qualitative findings and analysis

Theme	Findings	Supportive quote
**Feedback on the campaign and app**	Sugar cubes were an appropriate quantitative measure for target audience.	*“Yes, even though [children] actually don’t know how much is in a cube, but some of the things in cans in the shops they have like 36 cubes and she goes, “36!” and then it’s really worked wonders for us.”* (Parent1)
	The app was useful, fun and hands on for children to use.	*“This app is a really good idea because the younger generation are all obsessed with all these iPads and smartphones, so I guess that’s a good way for them to get a bit of new knowledge in their head”* (Parent2)
**Campaign messages and impact on sugars intake**	Parents engaged with the message to limit amount of sugars.	*“Look at what you’re eating. Look at how much added sugar is in the food that you’re buying and replace it with lower sugar”* (Parent3)
	The app helped parents make purchasing decisions when shopping.	*“I knew that fruit juices were high in their own sugars, but for him to see the amount of sugar in the carton of fruit juice, it made him go, “Oh, actually, yes”.”* (Parent3)
	The app prompted family discussions around food.	*“It helped springboard the conversation with them, and it helped as well to get the core message home about healthier eating and about managing sugar in your diet”* (Parent4) *“They were quite interested in going round the house to scan the foods, to see all the different sugars in the juices that they ask for and things like that that we always say, “There’s too much sugar.” They didn’t understand, so by seeing it on there it was quite interesting for them to see.”* (Parent12)
	The campaign instigated dietary changes through reducing portion size, changes to purchasing habits or substitution with healthier options.	*“The Sugar Smart App, that’s helped a lot, because my daughter likes cereal bars, but she’s gone off them now because of that App”* (Parent1) *“we found out about the [Brandname] yoghurt thing, because we picked that up and put that in the trolley and when it was scanned, it was like, “Yes, that can go back.” …*. *Yes, we didn’t even buy it in the end.”* (Parent5) *“There was the chocolate milk that had quite a lot of sugar in it. You’d go, “Okay, well, I thought it wouldn’t have been that good anyway because it’s chocolate milk but, okay, we’ll still buy it occasionally. Then, we’ll make sure that instead of you finishing the bottle, we’ll just put it in the glass and you have this much.” It’s about limiting and about being portion control aware”* (Parent3) *“The desserts have changed; actually, they have, yes. Whereas they used to go and want cake and custard all the time, then they do like fruit, so they tend to have more fruit for dessert and puddings, so that’s changed”* (Parent6)
	The campaign raised children’s awareness and dietary changes were made.	*“[My daughter] will sometimes say to me, ‘I’d best not have that; I’ve had too much sugar today already.’”* (Parent7) *“When he was eating his chocolate, he asked me, “How many sugar cubes in this chocolate?”* (Parent2)
	Some parents were critical of substitution with sweeteners.	*“That was one thing about the campaign, it does say that you can have sugar free drinks. I’m not sure that that’s necessarily right […*] *letting them develop a taste for something very sweet is not the way.”* (Parent8)
***Reported barriers to reducing sugar intake***	Parents criticised schools for promoting a ‘pudding culture’.	*“It seems silly. They should just offer a piece of fruit or nothing. I don’t know why there’s this thing about having pudding is still there, really […*] *it’s bizarre. It’s a very old fashioned kind of thing”* (Parent8) *“I am quite sure they are offered cake in school now. It is apple crumble and custard. This idea that you have to have a sweet thing after your dinner, it is like you have to have something.”* (Parent9)
	Parents described the existence of a ‘treat culture’: sugars-rich treats are easily available and are used to ‘bribe’ children to eat.	*“If we go to a shop they think they can just have a treat. Every time we are at a shop they are like, “Mum can we have a treat?””* (Parent9) *“As a parent it’s really easy to end up giving in to your kids, to reward your kids or pacify them with treats”* (Parent7)
	Misleading food marketing: Parents commented on misleading food marketing; in particular, dried fruit based snacks (also milkshakes, chocolate spreads, cereals, pasta sauces, cheese sticks, cordials and cereal bars).	“*Even those [snack bars] are really bad, because they’re just processed, and they just stick to your teeth like [sweet candies] which is the other problem, obviously, the tooth decay. So, even those, even though they count as one of your five a day and they’re marketed as healthy, they count as a sweet in our house …*” (Parent10) *“I had a habit of just buying those [processed fruit product], the presumption was, it’s only natural ingredients, it’s fruit-based, it’s fine, but then you scan it with the app and you think, “Wow, it’s got that much sugar in there,”* (Parent11)
	Parent’s busy lifestyle led to leniency with regard permitting sugar-rich treats.	*“You get tired and sometimes you go, “oh, just eat it then”* (Parent8)
	Parents expressed a reticence to deny children sugar-rich treats.	*“I like to think that we give the children a balanced diet anyway, so I think, especially with young children, you can’t get away from sugar, they absolutely love it, but who doesn’t? It’s just about having things in moderation, at least that’s what I’m trying to do”* (Parent4)
	Parents struggled with pressure from peers.	*It’s very hard when you’ve got friends of the family or friends of the children who go, “[name] is allowed a bottle of Coke at school,” or, “[name] takes a bottle of Coke.” … It’s hard to compare to other people”.* (Parent12)
	Parents exhibited unrealistic optimism regarding the relevance of the campaign to their child.	*“I haven’t made any changes, because I think, overall, our diet is pretty good”* (Parent13) *“she’s a very active child so I haven’t necessarily got any concerns over her diet”* (Parent14)
	Confusion which sugars to avoid and how to explain to their child the distinction between these sugars and those that are not considered bad for health (i.e. those sugars naturally present in whole fruits and vegetables and milk).	*“We scanned one of the Greek plain yoghurts and the machine said it had no added sugar, but, actually, on the label of that can, it said it had sugar.”* (Parent2) *“[The children] thought because they couldn’t have sugar, they couldn’t have fruit and it was saying that sugar was bad, so we had to sort of explain that it’s not all bad; it’s just different types.”* (Parent6) *“I used to do Weight Watchers so I’m savvy enough to know what vegetables have a higher concentration of sugar and to limit those and to have a mixture of vegetables … only have one piece of fruit a day and to have the other four vegetables to keep sugar down. So yes, that’s it”* (Parent15)

## Discussion

To the authors knowledge this is the first study to measure the impact of a health marketing campaign aimed at reducing sugars consumption on the detailed dietary behaviour of a national sample of children. The final sample was balanced for gender split, however, despite weighting the sampling procedure, the final sample included a relatively higher proportion of families from the ABC1 group. The study has shown that the health marketing campaign was successful in reducing the mean intake of total sugars by approximately 2% of total energy intake in a group of children whose families had shown an interest in previous Change4Life campaigns, however reductions were not sustained at the 12-month follow up. The qualitative findings indicate that parents want to reduce their child’s sugars intake but societal barriers and confusion over types of sugars hamper efforts to change. The unintentional increase in the mean amount and percentage of energy derived from dietary fat of between 1 and 2.4%, may have resulted from an increase in consumption of milk or of higher fat savoury snacks as replacements for sugars-containing items. This finding highlights the importance of giving any nutrient specific advice in the wider context of a healthy diet; indeed the PHE’s 2017 campaign focused on all elements of a healthier diet under the banner of Change4Life ‘Be Food Smart’ [26].

The reported data for intake of sugars and nutrients are comparable with the recently published UK data from the NDNS [12], which showed the average intake of Free Sugars was 54.5 g and 49.9 g for boys and girls aged 4–10 years respectively, contributing 13.6% (boys) and 13.4% (girls) to daily energy intake. The data are similar to that for added sugars from other industrialised countries including Australia and the US: data from the 2011–12 Australian Health Survey showed children aged 4–8 years obtained 11.9% of energy from added sugars (~ 50 g/ day) [27]. Data from the US NHANES for the 6–11 years age group showed added sugars (including honey and syrups) contributed 16.2% to energy intake [28].

The present study showed a wide range of values for the per capita median contribution of a food group, known to be high in sugars, to total sugars intake. This in part is explained by the wide age range of the study population, as the contribution of different foods to sugars intake will change with age. The sample size was, however, not large enough to allow meaningful sub-group analysis by age. The data show that soft drinks and fruit juice make relatively large contributions; a finding that concurs with data from the aforementioned surveys in the UK, Australia and the US [12, 27, 28]. What is distinct from the current data is that the median contributions from known sources of sugars did not account for total sugars intake. This suggests that children obtain a substantial proportion of sugars from less obvious sources i.e. foods not perceived by parents to be high in sugars. The qualitative data support this and indicate concern amongst parents about the amount of sugars in unsuspected items such as pasta sauces and some breakfast cereals.

The campaign made no notable impact on the contribution of dietary sources of sugars. Trends towards an increased contribution from soft drinks due to a possible shift in the source of sugars from foods to drinks, a trend which warrants further investigation, and a slight reduction in that from fresh fruit are of concern and contrast to the campaign messages. However, the qualitative data indicated confusion over ‘bad’ and ‘good’ sugars, with reference being made to restricting fruit intake because of sugars content. Despite the Sugar Smart app including clear information with respect to which sugars to limit, this sound information competes with misleading misinformation from other sources such as the media and internet. The public need to be made aware of where to look for credible information on food and nutrition, including information on sugars.

The qualitative findings showed that the health marketing campaign raised awareness of sugars in foods and drinks in both parents and children and impacted on foods bought and consumed by families. Data showed that in general, parents supported the messages of the campaign and want to control their child’s sugars intake, but face challenges in putting this into practice, including misleading marketing, less obvious sources of sugars and confusion for which types of sugars to reduce.

The study has several limitations. First, parents were selected from the PHE Change4Life database, therefore it could be argued that they were potentially more motivated to make changes to their children’s diets. Measuring individual exposure to the campaign was not possible. Second, food and drink intake was assessed at weekends only. This was because children were not of an age where they could reliably record their own food intake and parent records of child food intake had to be relied upon. Recording at a weekend, when giving treats may be more likely, may have in part obscured the impact of the campaign. Children under the age of 10–11 years are unlikely to be able to accurately recall their food and drink intakes and estimate portion sizes due to limited cognitive ability [29]. Although conducted in a younger age group, research by Wallace et al. (2018) found that parents of pre-school children were able to report the food and drinks their child consumed using an online 24-h recall tool similar to Intake24 with reasonable accuracy [30]. Thirdly, the PHE Change4Life campaign was scheduled to run immediately in the New Year, following the holiday period as this is a time when people may be more perceptive to making healthy lifestyle changes [31]. This meant that seasonal activities may have impacted upon baseline data and seasonal fluctuation in intake may have contributed to the changes observed at peak campaign. To explore this phenomenon and control for seasonal variation, long-term follow up included data collection exactly 12 months from baseline and data at 10 month post baseline (outside the Christmas season). Although our results suggest a decrease in sugars intake that was not sustained, there were differences between 10 and 12 months data. At 10 months post-campaign there was a statistically significant decrease in percentage energy from Free Sugars, but this was not sustained at the 12 month follow up. Therefore a seasonal influence on sugars intake cannot be ruled out. Finally, the study relied on self-reported dietary data, the limitations of which, including recall bias, are discussed elsewhere [32].

## Conclusions

The Sugar Smart health marketing campaign was successful in raising awareness of sugars intake among parents and children who had shown an interest in previous Change4Life campaigns. This study suggests the campaign had a positive impact in reducing sugars intake, however reductions in sugars were not sustained. The findings suggest improved consumer education on different types of sugars and improved food labelling are needed. The health marketing campaign was, however, only one of a broad range of measures being introduced collectively by PHE to reduce sugars intake.

## Data Availability

The datasets used and/or analysed during the current study are available from the corresponding author on reasonable request.

## Abbreviations

NDNS: National Diet and Nutrition Survey
NHANES: National Health and Nutrition Examination Survey
NMES: non-milk extrinsic sugars
PHE: Public Health England
SACN: Scientific Advisory Committee on Nutrition

## Footnotes

1 The sugars naturally present in milk and dairy products, fresh and most types of processed fruit and vegetables and in cereal grains, nuts and seeds are excluded from the definition.
